# Evaluating base and retrieval augmented LLMs with document or online support for evidence based neurology

**DOI:** 10.1038/s41746-025-01536-y

**Published:** 2025-03-04

**Authors:** Lars Masanneck, Sven G. Meuth, Marc Pawlitzki

**Affiliations:** Department of Neurology, Medical Faculty and University Hospital Düsseldorf, Heinrich Heine University Düsseldorf, Düsseldorf, Germany

**Keywords:** Neurological disorders, Translational research

## Abstract

Effectively managing evidence-based information is increasingly challenging. This study tested large language models (LLMs), including document- and online-enabled retrieval-augmented generation (RAG) systems, using 13 recent neurology guidelines across 130 questions. Results showed substantial variability. RAG improved accuracy compared to base models but still produced potentially harmful answers. RAG-based systems performed worse on case-based than knowledge-based questions. Further refinement and improved regulation is needed for safe clinical integration of RAG-enhanced LLMs.

## The promise and pitfalls of large language models in making information accessible

While neurological conditions are the leading cause of disease burden worldwide^1^, neurology’s broad scope and complexity^2^ drive increasing subspecialization^3^. Large language models (LLMs) – probabilistic artificial intelligence systems that generate human-like text – have gained attention due to their versatile applications across various industries, including medical research and healthcare^4^. Products such as ChatGPT or its underlying models have been shown to pass medical exams^5^, support patient communication^6^ or consent^7^, and achieve remarkable results in diagnosis or triage^8–10^. While LLMs often outperform simple online searches for differential diagnosis, their performance currently still lacks accuracy^11^. This is often attributed to the probabilistic nature of the models, which results in limited reasoning capabilities and hallucination phenomena^4^. One advocated solution is retrieval-augmented generation (RAG), combining an LLM with a searchable knowledge store to guide its answers^12^. The knowledge store can consist of diverse sources, such as a static document repository or a dynamic resource like web search. LLMs using RAG have shown first promising results in the medical domain^13,14^.

As clinical fields such as neurology deal with increased specialization and a growing knowledge base, LLMs may help clinicians by making up-to-date information readily available, including beyond their subspecialty. In this study we investigated the extent to which LLMs, with or without RAG, can provide guideline-adhering answers to practically relevant neurological questions with appropriate source attribution. Seven advanced base models (both open-source and proprietary) were tested, alongside one model using a fixed RAG setup with a document store of relevant guidelines, and another incorporating web search-enabled RAG capabilities. We created 130 questions (Supplementary Table 1) – half knowledge-based and half hypothetical case-based – derived from 13 current American Academy of Neurology (AAN) guidelines (Supplementary Table 2) spanning neuroimmunology, infectious diseases, epilepsy, movement disorders, neurovascular disease, headache disorders, polyneuropathies, brain death, sleep disorders and tic disorders.

## Assessment of LLM performance

**Fig. 1:**
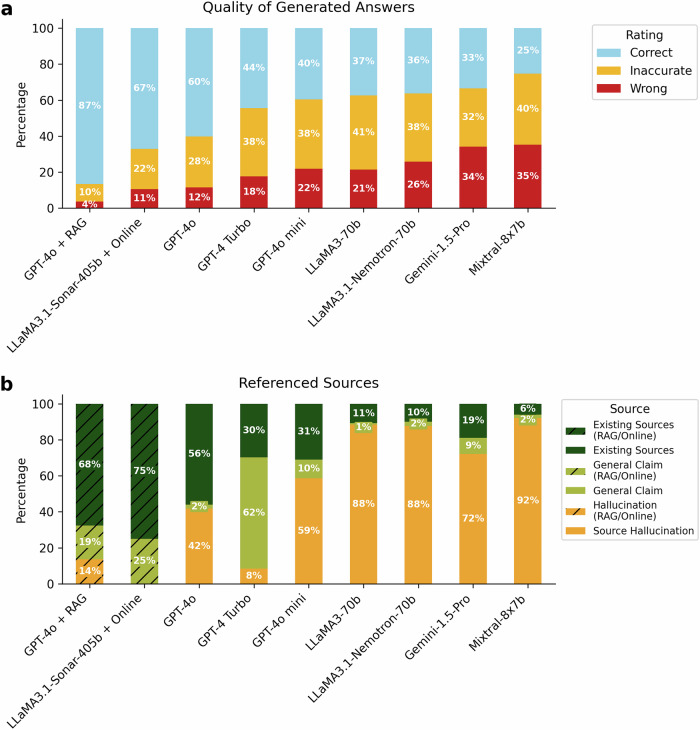
Quality of LLM responses to practically relevant neurological questions compared to AAN guidelines. **a** Stacked bar plot displaying the percentage of responses for each rating category as evaluated by neurologists according to the respective American Academy of Neurology (AAN) guideline. Categories include “Correct” (blue; fully aligned with the guidelines), “Inaccurate” (yellow; containing minor errors), and “Wrong” (red; substantially incorrect, dangerous or misleading). **b** Stacked bar plot illustrating the categorization of referenced sources in LLM responses, verified through bibliographic and web searches. Categories are: ‘Existing Sources’ (dark green), ‘General Claims’ (light green; no specific source mentioned), ‘Source Hallucination’ (yellow), with document or online retrieval-augmented generation (RAG) enhancements marked by hatch lines including ‘RAG/Online + Existing Sources’, RAG/Online + General Claim’, and RAG/Online + Hallucination’. All models were queried 130 questions (half case-based, half knowledge-inquiring) four times, thus the percentage is respective to 520 total answers. Due to rounding, totals may not sum to exactly 100%.

All tested base models (GPT-4o, GPT-4 Turbo, GPT-4o mini, LLaMA3-70b, LLaMA3.1-Nemotron-70b, Gemini-1.5 Pro, and Mixtral-8x7b) as well as the RAG-enabled systems (GPT-4o with document RAG (document-RAG GPT-4o) and LLaMA3.1-Sonar-405b with online RAG (online-RAG LLaMa3.1) (Supplementary Table 3 for details) delivered 520 responses across four iterations of 130 questions. Inter-rater agreement of LLM responses between the primary raters showed a high consistency of ratings with a Cohen’s kappa of 0.915. Mixtral-8x7b provided the fewest correct answers (131; 25%), followed by Gemini-1.5 Pro (174; 33%). The group of LLaMA3.1-Nemotron-70b, LLaMA3-70b and GPT-4o mini showed comparably similar and slightly better performance, with 189 (36%), 194 (37%), and 206 (40%) correct answers, respectively. Among best-performing base models, GPT-4 Turbo produced 231 (44%) correct, 197 (38%) inaccurate, and 92 (18%) incorrect answers, while GPT-4o outperformed it with 313 (60%) correct, 147 (28%) inaccurate, and 60 (12%) incorrect. With RAG support, document-RAG GPT-4o reached 450 (87%) correct, 51 (10%) inaccurate and 19 (4%) wrong answers. The online-RAG LLaMa3.1 showed intermediate performance in between base models and the document-RAG GPT-4o, achieving 349 (67%) correct, 116 (22%) inaccurate and 55 (11%) wrong answers (Fig. 1a).

Statistical analysis of the modal rating for each question revealed significant differences (*P* < 0.001) between models. Pairwise testing confirmed the document-RAG GPT-4o to be performing significantly better than all others, followed by the online-RAG system and the base GPT-4o model, with no significant difference between both. Statistical differences between the further base models were gradual with detailed results of the pairwise comparisons depicted in Supplementary Fig. 1 and listed in Supplementary Table 4. Computer-assessed linguistic features showed higher cosine similarity of responses to example answers in better-performing models, though its usefulness was limited by the varying formats of LLM answers (Supplementary Table 5).

Across models, few responses contained correct sources (e.g., suitable scientific papers). Some frequently included faulty or fabricated citations: Mixtral-8x7b fabricated sources in 479 responses (92%), while some models like GPT-4 Turbo often made general claims - like “according to AAN” - in 321 responses (62%), without specifying sources. The best-performing base model, GPT-4o, more often cited exclusively existing sources (291 responses, 56%) but fabricated sources in 217 (42%) responses. document-RAG GPT-4o improved correct citations to 352 (68%), with 71 (14%) fabrications, while online-RAG LLaMA3.1 had only one source hallucination and cited sources correctly in 390 (75%) answers (Fig. 1b).

RAG-based systems also performed better on knowledge-based than case-based questions (document-RAG GPT-4o: 92% versus 82% correct answers; online-RAG LLaMA3.1: 72% versus 63%). In contrast, base models showed smaller differences, except for Mixtral-8x7b, which performed better on cases (19% versus 31%) (Supplementary Fig. 2). Ordinal logistic regression confirmed these trends, with significant odds ratios (ORs) favoring knowledge-based questions for document-RAG GPT-4o (OR = 1.85, *P* < 0.001). Although online-RAG LLaMA3.1 displayed a similar trend, it was not significant after correction for multiple testing (*P* = 0.013). Conversely, Mixtral-8x7b favored case-based questions (OR = 0.58, *P* = 0.03) (Supplementary Table 6).

When analyzing performance and source attribution over time, one could generally observe guideline-specific differences. While overall performance was very high in certain guidelines from 2022 and 2023 (e.g., brain death and neurovascular guidelines), base GPT-4o’s performance tended to drop on newer guidelines whereas online-RAG LLaMA3.1 improved. A spike in source hallucination was observed for the 2024 guideline, likely due to its exclusion from the training data (Supplementary Figs. 3 and 4). Models also differed in consistency when repetitively asked the same questions: Gemini-1.5 Pro produced differently rated responses for 50 of 130 questions, while LLaMA3.1-Nemotron-70b varied only three times (Supplementary Fig. 5). RAG reduced variability, as the document-RAG GPT-4o showed fewer varying responses compared to its base counterpart (15 versus 33).

## Clinical relevance and pathways for future integration

This study evaluated advanced LLMs, including ChatGPT-related models, for providing evidence-based guidance aligned with current AAN guidelines. Performance varied, and all models occasionally produced incorrect, outdated, or potentially harmful responses. Errors included reliance on outdated guidelines, ambiguous procedural details and incomplete retrieval of relevant information (Table 1). Despite increasing use in professional medical contexts, LLMs cannot yet reliably generate accurate, guideline-based answers for specific neurological issues. Furthermore, the results expose the limited reliability of sourced references, with the majority containing incorrect or fabricated bibliographic information. This issue, likely stemming from the probabilistic nature of LLMs, limits the practical use of such technologies for further medical engagement.

**Table 1:** Common error types in LLM and RAG pipelines

Error type	(I) Pipeline Location: LLM	(II) Pipeline Location: RAG	Examples
(A) Outdated/ Conflicting Information	Draws on stale/incorrect data from internal training or hallucinates older guidelines.	Retrieves and merges older or contradictory external sources over more recent, correct guidelines.	(I) Answer refers to a 2010 brain death protocol that no longer aligns with current AAN guidance; (II) LLM merges contradictory guidelines for PFO workup.
(B) Missing Clear Cutoffs	Invents or confuses numeric thresholds (e.g., dosage, temperature).	Retrieves documents with wrong cutoff values and fails to reconcile them with accurate data.	(I) States 32°C instead of 36°C for brain death temperature requirement. (II) Repeats an outdated snippet about dosage thresholds from a retrieved document.
(C) Ambiguous or Contradictory Statements	Provides multiple recommendations without unifying them, creating unclear or contradictory steps.	Pulls partial or mismatched instructions from retrieved documents, failing to generate a coherent conclusion.	(I) Suggests serological testing but also a lumbar puncture without explaining why or how. (II) Retrieves conflicting neuroborreliosis guidelines and doesn’t clarify a unified approach.
(D) Misleading or Distorted Context	Correct fact overshadowed by faulty framing (e.g., labeling correct advice as non-compliant).	Combines incomplete or conflicting sources in a way that misrepresents otherwise correct guidance.	(I)&(II): Correctly identifies valproate as most teratogenic but pairs it with outdated folic acid info, confusing the overall recommendation.
(E) Substantially Faulty Rationale (Despite Correct Conclusion)	Delivers the right answer but uses an incorrect or misleading explanation.	Integrates the right source but misreads or merges its rationale incorrectly.	(I)&(II): LLM answer offers correct folic acid dosage but claims epilepsy guidelines don’t address this (which is untrue).
(F) Omission of Critical Details	Fails to incorporate key question or case facts, producing incomplete or off-target recommendations.	Misunderstands task and retrieves faulty document or fails to report essential details from correct source after retrieval.	(I) Overlooks spontaneous breathing in a brain death case and cites an irrelevant apnea test. (II) Retrieves the right guideline but omits mention that a recommended treatment is off-label.
(G) Prompt Misinterpretation or “Non-Answer”	The model misunderstands the user query or sidesteps the question entirely.	Does not properly process (correctly) retrieved document.	(I) Summarizes guidelines rather than answering the user’s specific query. (II) Answer states that “no further input is needed” because the retrieved guideline “looks good,” never providing the requested specifics.

This table outlines seven observed recurring error types in large language models (LLMs). Column (I) shows how these errors arise solely within the LLM; Column (II) highlights how retrieval-augmented generation (RAG) can introduce or amplify similar issues. Example scenarios illustrate each pitfall in clinical contexts.

However, substantial improvements in response quality and source reliability emerged when LLMs had access to relevant data, as with our RAG setup embedding AAN guidelines in a vector database or the web search-aided model. Given the expanding complexity of medical fields like neurology, LLMs enhanced with techniques like RAG could provide more reliable, swifter and more comprehensive access to essential knowledge contained within guidelines or other relevant sources. RAG also likely mitigates the issue of fast turnover time of medical information, which is illustrated by the performance drop of base models for a 2024 guideline, which was likely not included in training data. By facilitating easier access to, and possibly educating on, standards for a broad spectrum of indications, such systems have the potential to enhance the availability of evidence-based information and may help in cultivating diagnostic and therapeutic skills among clinicians. While RAG-enabled systems performed better, they still made errors that were highly dependent on the information retrieved, particularly for the web search-aided system. Notably, RAG-enabled systems performed worse on case-based questions compared to knowledge-inquiring questions, likely due to less similar wording that influenced the precision of retrieval. This highlights an important feature of these systems, which perform better in abstracted scenarios with the correct vocabulary present, further highlighting the need for rigorous, balanced and diverse testing. The document-based RAG setup demonstrated generally better performance, though this may be slightly biased since it was restricted to the exact guidelines it was tested on, whereas the online-based system is designed for universal application. Both systems have significant room for improvement. For instance, the online-based system could be enhanced by whitelisting specific domains, thereby restricting sources to authoritative web addresses (e.g., the AAN website). The online RAG-based system also appeared to improve for more recent guidelines, highlighting a recency bias of web search that might be addressed.

Widely used comparisons of LLM capabilities (e.g., lmarena.ai^15^), inadequately predicted performance on guideline-adherent answers as top-ranked open-source (LLaMA3.1-Nemotron-70b) and proprietary models (Gemini-1.5-Pro) performed relatively poor. Several factors could explain this discrepancy, including model-specific prompt requirements, which should be carefully considered when using such systems for domain-specific tasks, such as in medicine. Overall, GPT-4o outperformed other models and no significant differences were observed between proprietary and open-source models, a relevant point given the varying application scenarios and associated privacy considerations.

While these systems show potential, further improvements are crucial. Key considerations include determining an acceptable error level for clinical use, if any. And while first guidelines and regulations are established^16,17^, the extent and circumstances under which clinicians can rely on this information remain to be defined. These systems need to be tested in further rigorous validation studies, while regulatory pathways to integrate such technologies into clinical practice need to be defined.

## Methods

Sufficiently detailed AAN guidelines, which included recommendations, and were newly published within the last five years (after May 1^st^, 2019), were selected for analysis. Thirteen guidelines spanning various neurological topics qualified for inclusion (Supplementary Table 2). The authors, all practicing neurologists, selected five clinically relevant aspects for each guideline and designed two questions per aspect: one hypothetical case-based and one knowledge-based (see Supplementary Table 1 for questions and sample response guidance). Due to the probabilistic nature of LLMs, these questions were posed four times each to the base LLMs GPT-4o, GPT-4o mini, GPT-4 Turbo, LLaMA3-70b, LLaMA3.1-Nemotron-70b-instruct, Gemini-1.5 Pro, and Mixtral-8x7b. At the time of most recent querying in November 2024 these models included the best-performing proprietary and open-source models according to online comparisons^15^. All models were prompted to answer based on evidence-based guidelines from trusted sources like the AAN, include details and list sources (see Supplementary Note 1 for the previously iterated zero-shot prompt and Supplementary Table 3 for model parameters, knowledge cutoff dates and model details).

Two blinded raters (4 and 9 years of neurological experience as doctors) assessed the responses for accuracy, categorizing them as “correct,” “inaccurate,” or “wrong” based on guideline recommendations, with a third rater (20 years of practical neurological experience as a doctor) resolving any disagreements. Raters were instructed to classify an answer as “correct” if the recommendation itself was entirely accurate, even if minor, inconsequential errors in the reasoning process were present, as long as these did not impair understanding or cause potential clinical consequences. Responses were labeled “inaccurate” if they were incomplete, contained minor errors, or had illogicalities that could lead to a misunderstanding of an otherwise generally correct answer. Responses deemed “wrong” did not answer the question or contained incorrect, highly incomplete, or potentially dangerous information. The assessment prioritized accordance with the current evidence-based standard and safety, without considering references or sources (see also the dataset with ratings in the online repository and a set of simplified examples in Supplementary Note 2). The additionally rated reported sources were independently evaluated by one rater to determine whether they were general claims, correctly cited, or at least partially fabricated (“hallucinated” – used here for consistency with literature, though it more accurately reflects a neurological confabulation). This evaluation was conducted by searching the presented bibliographic information via web search, digital object identifier (DOI), and Pubmed/Medline. Sources were considered correct only if all information, including bibliographical details and DOI, was accurate. Further, the reported source was required to contain at least partially helpful information to the question. If one source in a response was ‘hallucinated’, the entire response was categorized likewise.

**Fig. 2:**
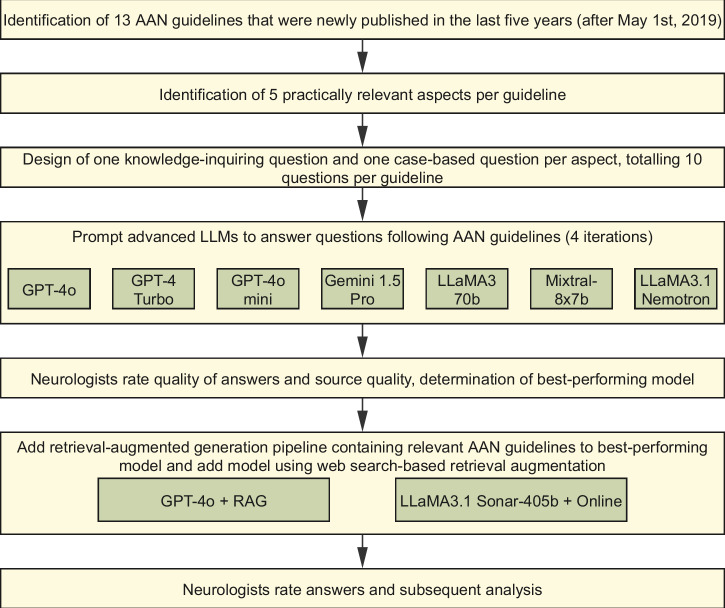
Flowchart of the study’s different chronological steps. A brief overview of the study design. American Academy of Neurology (AAN), large language model (LLM), retrieval-augmented generation (RAG).

The top model underwent further testing with a RAG setup embedding guidelines in a searchable vector database to enhance accuracy. The document RAG setup is based on the setup used by other authors^14^ and is available in its implementation along with all used code here: https://github.com/Entspannter/LLMs-RAG-Neurology. A second RAG setup employed the online-accessible LLaMA3.1-Sonar-405b model, which incorporates web search results into its responses^18^. A flow chart of the study can be found in Fig. 2.

Statistical evaluation of the response quality was conducted using the non-parametric Friedman test to compare response accuracy across models, with subsequent non-parametric two-sided Wilcoxon signed-rank tests for pairwise comparisons of the numerically encoded ratings. For these analyses, the modal rating for each question for each model was used. To compare the performance of models in case-based and knowledge-based questions, ordinal logistic regression was applied. For both analyses, P-values were adjusted for multiple comparisons using Bonferroni correction. For exploratory and objective analysis of the LLM responses, cosine similarity and BLEU (bilingual evaluation understudy) scores were calculated by comparing the LLM outputs with simplified, guideline-coherent example answers. Percentages were rounded to the nearest whole number.

## Supplementary information

Supplementary Materials

## Data Availability

The data assessed in this study can be found in the respective GitHub repository https://github.com/Entspannter/LLMs-RAG-Neurology/.

## Code availability

The underlying code is available at the following GitHub repository: https://github.com/Entspannter/LLMs-RAG-Neurology/.
