# Supplementary material for: Evaluating base and retrieval augmented LLMs with document or online support for evidence based neurology
Source: NPJ Digit Med. 2025 Mar 4;8:137. doi: 10.1038/s41746-025-01536-y (PMC11880332; doi:10.1038/s41746-025-01536-y)

Supplementary Note 1
Depending on the exact model application programming interface (API), the following prompt was either put in as “system” message (OpenAI API, Mixtral, LLaMA, Nvidia API, Perplexity API) and followed by the question as “user” message” or the question followed the prompt directly (Gemini).

Prompt:

“*You are a dedicated AI medical assistant specializing in neurology. Your answers should be based on medical guidelines such as the most recent AAN guidelines and are strictly truthful. If the information is not available in guidelines, state it clearly. Refrain from including irrelevant or out-of-context details. Please respond to the following question using information from guidelines. Focus on providing answers that are:*

- Directly linked to the guidelines, citing specific sections or page numbers when possible. Make sure to mention the guidelines you are referencing and the source.

- Presented in concise bullet points. Do not bloat the answer unnecessarily. Keep it short and professional.

- Comprehensive, including all relevant details about disease type, disease stage, patient age, and clinical trials when applicable.

- Honest, clearly stating if the information is not covered in the guidelines.

- Including citations to the guidelines or other reputable sources as needed.

Based on guidelines such as the most recent AAN guidelines, provide a detailed and truthful response that addresses the specifics of the question. Ensure all relevant medical and scientific details are included to support your answer.”

Supplementary Note 2
Simplified Example Answers for the Different Rating Category

Question 34: According to current guidelines, should EEGs, AEPs, or SEPs be used as ancillary tests to assist with the diagnosis of Brain Death?

Correct: There is a clear recommendation against using these ancillary tests.

Inaccurate: EEG is not recommended. AEPs and SEPs are not mentioned in the guideline, but generally the AAN recommends a clinical examination following a predefined protocol.

Wrong: AEPs and SEPs are not recommended, EEG, however, is recommended.

Supplementary Table 1: AAN Guidelines and Respective Questions

Guideline	Question Number	Question Type	Question	Answer According to Guideline
**Practice advisory update: Antiseizure medication withdrawal in seizure-free patients**	1	Knowledge	According to current guidelines, is there statistical evidence to support the discontinuation of antiseizure medications (ASMs) in an adult epilepsy patient who has been seizure-free for more than two years?	While there is a possibly higher seizure recurrence rate in patients with ASM withdrawal and there is a small chance that ASM will not work in the same way after withdrawal, there is no statistically significant evidence to support either option. There is particularly no strong evidence regarding status epilepticus and death after ASM withdrawal.
	2	Knowledge	According to current guidelines, is there evidence for EEG or imaging to support the decision to discontinue antiseizure medications (ASMs) in adult epilepsy patients?	There is no evidence for either - currently the recommendation is that patients should be informed that the relevance of both is unknown. There is very limited evidence in children for the EEG modality.
	3	Knowledge	According to current guidelines, is there reliable data on the risk of seizure recurrence with antiseizure medications (ASMs) withdrawal in patients who have had epilepsy surgery and are seizure free?	There is only limited low-quality data and guidelines recommend clinicians to discuss that it currently is uncertain due to lack of evidence.
	4	Knowledge	According to current guidelines, is there evidence for EEG to support the decision to discontinue antiseizure medications (ASMs) in children with epilepsy that have been seizure-free for two years?	For children there is limited confidence that EEG abnormalities predict the recurrence of seizures in children. The guidelines recommend to order an EEG if ASM withdrawal is a possibility in the specific case. Withdrawal is only recommended as option if an EEG does not show an epileptoform activity.
	5	Knowledge	According to current guidelines, is there is strong evidence regarding the relationship between antiseizure medications (ASMs) withdrawal and changes in the risk of mortality and status epilepticus in patients with epilepsy that have been seizure free for several years?	No, there is not.
	6	Case	A 34-year old man is on antiseizure medication and has been seizure free for 7 years. He is discussing with his doctor whether to stop his antiseizure medication or not. According to current guidelines, is there evidence that an EEG can inform this decision?	There is no evidence for either - currently the recommendation is that patients should be informed that the relevance is unknown.

	7	Case	A 9-year-old girl has been treated for seizures for 4 years and her parents want to discuss the withdrawal of antiseizure medication with their neurologist. Is there evidence that an EEG can inform this decision?	For children there is limited confidence that EEG abnormalities predict the recurrence of seizures in children. The guidelines recommend to order an EEG if ASM withdrawal is a possibility in the specific case. Withdrawal is only recommended as option if an EEG does not show an epileptoform acitivity.
	8	Case	A 57-year-old patient with epilepsy has been seizure-free on levetiracetam for several years. He wonders whether there is proof, according to current guidelines, that withdrawing his medication puts him at risk for earlier death in this scenario.	There is no evidence for this and the guidelines state this.
	9	Case	A 26-year-old female patient with a history of generalized seizures is considering pausing her medication after several years of being seizure-free. She wonders whether new imaging might support this decision, according to current guidelines. Previous imaging was without findings.	There is no evidence for renewed imaging according to the current guideline.
	10	Case	A 33-year-old male had several generalized seizures in his early twenties and has been seizure-free for seven years while undergoing treatment with lamotrigine. He wonders whether there are common recommendations (e.g., in a guideline) to support continuation or discontinuation of his medication.	While there is a possibly higher seizure recurrence rate in patients with ASM withdrawal and there is a small chance that ASM will not work in the same way after withdrawal, there is no statistically significant evidence to support either option. There is particularly no strong evidence regarding status epilepticus and death after ASM withdrawal.
**Teratogenesis, perinatal, and neurodevelopmental outcomes after in utero exposure to antiseizure medication: Practice guideline from the AAN, AES, and SMFM**	11	Knowledge	According to current guidelines, what antiseizure medication is most commonly linked to neural tube defects?	The guideline states: "Valproic acid is associated with the highest unadjusted birth prevalence of neural tube defects (1.4%) as compared to other ASMs."
	12	Knowledge	According to current guidelines, should providers definitely switch an epilepsy patient that becomes pregnant from a medication with a known risk for the fetus to another antiseizure medication when the epilepsy is well-controlled under the medication?	No, there is no definitive recommendation as this could also increase the risk of status epilepticus and similar during pregnancy. The two relevant recommendations are the following: 2A. Clinicians must minimize the occurrence of convulsive seizures (generalized tonic-clonic seizures and focal to bilateral tonic-clonic seizures) in patients with epilepsy during pregnancy to minimize potential risks to the birth parent (e.g., seizure-related mortality) and to the fetus (Level A).<br>2B. Once a patient with epilepsy is already pregnant,

				clinicians should exercise caution in attempting to remove or replace an ASM that is effective in controlling generalized tonic-clonic or focal to bilateral tonic-clonic seizures, even if it is not an optimal choice with regards to the risk to the fetus (e.g., valproic acid) (Level B).
	13	Knowledge	According to current guidelines, is phenobarbital a comparably safe choice in potentially pregnant women or does it have a known higher risk for certain malformations compared to other antiseizure medications?	It should be avoided if possible as it has an increased risk for cardiac malformations.
	14	Knowledge	According to current guidelines, should folic acid be supplemented in all epilepsy patients that become pregnant?	Yes, it is recommended (at least 0.4mg daily) both preconceptionally and during pregnancy and counseling for adherence is also recommended.
	15	Knowledge	According to current guidelines, what would be preferred antiseizure medications for epilepsy patients planning to become pregnant?	Clinicians must consider lamotrigine, levetiracetam, or oxcarbazepine if feasible.
	16	Case	A 22-year-old female patient with epilepsy is currently in the process of optimizing her medication together with her treating neurologist. According to current guidelines, which anticonvulsant is most likely to cause neural tube defects if she were to become pregnant while continuing treatment with the drug?	The guideline states: "Valproic acid is associated with the highest unadjusted birth prevalence of neural tube defects (1.4%) as compared to other ASMs."
	17	Case	A 33-year-old female patient with epilepsy has had several seizures and has just learned that she is pregnant while undergoing therapy with a potentially teratogenic medication. The epilepsy is currently well-controlled. Should she definitely switch the anticonvulsant according to current guidelines?	No, there is no definitive recommendation as this could also increase the risk of status epilepticus and similar during pregnancy. The two relevant recommendations are the following: 2A. Clinicians must minimize the occurrence of convulsive seizures (generalized tonic-clonic seizures and focal to bilateral tonic-clonic seizures) in patients with epilepsy during pregnancy to minimize potential risks to the birth parent (e.g., seizure-related mortality) and to the fetus (Level A).<br>2B. Once a patient with epilepsy is already pregnant, clinicians should exercise caution in attempting to remove or replace an ASM that is effective in controlling generalized tonic-clonic or focal to bilateral tonic-clonic seizures, even if it is not an optimal

				choice with regards to the risk to the fetus (e.g., valproic acid) (Level B).
	18	Case	A 30-year-old female patient with epilepsy, well-controlled on phenobarbital, is planning to start a family. She is concerned about the potential risks associated with continuing her current medication during pregnancy. Are there specific malformation risks linked to phenobarbital compared to other antiseizure medications according to current guidelines?	It should be avoided if possible as it has an increased risk for cardiac malformations.
	19	Case	A 28-year-old female patient with epilepsy, currently planning a pregnancy, asks whether she should take folic acid as a supplement. Her current treatment regimen includes antiseizure medication, and she is seeking advice on whether supplementation is recommended for all women with epilepsy who wish to become or already are pregnant, according to current guidelines.	Yes, it is recommended (at least 0.4mg daily) both preconceptionally and during pregnancy and counseling for adherence is also recommended.
	20	Case	A 27-year-old female patient with well-controlled epilepsy is planning to conceive. She is currently on antiseizure medication and asks her neurologist which medications would be preferable during pregnancy to minimize risk to the baby, based on current guidelines. What would be the best approach to adjust her treatment in preparation for pregnancy?	Clinicians must consider lamotrigine, levetiracetam, or oxcarbazepine if feasible.
**Stroke Prevention in Sympto Clinical Practice Guidelines by the Infectious Diseases Society of America (IDSA), American Academy of Neurology (AAN), and American College of Rheumatology**	21	Knowledge	According to current guidelines, should asymptomatic patients be tested for borrelia burgorferi exposure following a tick bite?	There is a strong recommendation against testing asymptomatic patients after a tick bite.
	22	Knowledge	According to current guidelines, are laboratory tests recommended in a patient with a history of a recent tick bite in a Lyme disease endemic area and currently presenting with erythema migrans?	Current guidelines recommend a clinical diagnosis in this case and do not require further laboratory tests. Laboratory tests are suggested for atypical lesions that are suggestive of erythema migrans.
	23	Knowledge	According to current guidelines, what is the recommended treatment for a patient with a history of a recent tick bite in a Lyme disease endemic area and currently presenting with erythema migrans?	Doxycycline, amoxicillin, or cefuroxime axetil - azithromycine as backup in case of allergies.

**(ACR): 2020 Guidelines for the Prevention, Diagnosis and Treatment of Lyme Disease**	24	Knowledge	According to current guidelines, in a patient with suspected neuroborreliosis, should a bacterial culture of cerebrospinal fluid be performed?	The current guideline recommends serum antibody testing in this case. In case that CSF antibody testing is instead carried out it recommends to test in the serum at the same time to calculate the index. There is a clear recommendation against CSF cultures.
	25	Knowledge	In a patient with suspected neuroborreliosis, should cerebrospinal fluid serology be carried out over serum serology?	No, serum antibody testing is preferred. If CSF antibody testing is done, it should always be carried out simultaneously with serum testing.
	26	Case	A 35-year-old male presents to his general practitioner after a recent tick bite. He is currently asymptomatic but is concerned about the potential risk of Lyme disease. He wonders if he should be tested for Borrelia burgdorferi exposure, given that he has not developed any symptoms. What would be a suitable apporach in accordance with current guidelines?	There is a strong recommendation against testing asymptomatic patients after a tick bite.
	27	Case	A 40-year-old female presents to her physician after noticing a red, expanding rash at the site of a recent tick bite. She recalls that the tick bite occurred about two weeks ago while hiking in a Lyme disease endemic area. The rash is consistent with erythema migrans, but she has no other symptoms. Should laboratory tests be conducted to confirm Lyme disease diagnosis according to current guidelines?	Current guidelines recommend a clinical diagnosis in this case and do not require further laboratory tests. Laboratory tests are suggested for atypical lesions that are suggestive of erythema migrans.
	28	Case	A 45-year-old male patient presents to his primary care physician with an expanding red rash consistent with erythema migrans, following a recent tick bite sustained while hiking in a Lyme disease endemic area. He is otherwise asymptomatic but is concerned about the appropriate course of action. What treatment approach should be taken based on current guidelines? What are second-line approaches?	Doxycycline, amoxicillin, or cefuroxime axetil - azithromycine as backup in case of allergies.
	29	Case	A 52-year-old male presents with symptoms suggestive of neuroborreliosis, including facial nerve palsy and headache. He reports a history of erythema migrans at the site of a tick bite approximately two months ago, which resolved	The current guideline recommends serum antibody testing in this case. In case that CSF antibody testing is instead carried out it recommends to test in the serum at the same time to calculate the index. There is a clear recommendation against CSF cultures.

			without specific treatment. A lumbar puncture is performed to obtain cerebrospinal fluid for analysis. The neurologist considers different diagnostic tests to confirm the diagnosis. Should a bacterial culture of the cerebrospinal fluid be performed in this case, according to current guidelines for suspected neuroborreliosis?	
	30	Case	A 45-year-old male presents with symptoms suggestive of neuroborreliosis, including persistent headache and radicular pain. He reports having had erythema migrans at the site of a tick bite about three months ago, which resolved spontaneously. A lumbar puncture is planned to obtain cerebrospinal fluid for further analysis. The physician needs to decide on the most appropriate diagnostic test. Should cerebrospinal fluid serology be performed instead of serum serology in this case, according to current guidelines for suspected neuroborreliosis?	No, serum antibody testing is preferred. If CSF antibody testing is done, it should always be carried out simultaneously with serum testing.
**Pediatric and Adult Brain Death/Death by Neurologic Criteria Consensus Guideline**	31	Knowledge	According to current guidelines, can brain death be diagnosed in patients younger than 37 week gestational age?	No, according to current guidelines it cannot and should not be determined in such patients.
	32	Knowledge	According to current guidelines, a provider's patient is spontaneously breathing but shows no other sign of brainstem function. Can he or she proceed to diagnose brain death in this patient?	No, as this shows brainstem or brain function the patient must not undergo brain death testing.
	33	Knowledge	According to current AAN guidelines, what is the minimum core temperature that should be reached before a patient can be diagnosed with brain death?	According to current guidelines, the minimum core body temperature should be 36°C. For patients that had a lower temperature previously it should be in place for more than 24h before initiating brain death assessment.
	34	Knowledge	According to current guidelines, should EEGs, AEPs, or SEPs be used as ancillary tests to assist with the diagnosis of Brain Death?	There is a clear recommendation against using these ancillary tests.
	35	Knowledge	According to current guidelines, should CT angiography, MRI angiography or conventional 4-vessel catheter angiography be used as ancillary tests to assist with the diagnosis of brain death?	Neither CT- nor MRI-angiography should be used. However, 4-vessel catheter angiography may be used according to the current guideline.

	36	Case	A preterm infant, born at 34 weeks gestational age, suffered a severe hypoxic-ischemic event resulting in significant neurological compromise. The medical team is considering the evaluation for brain death. Given the infant's gestational age, the neonatologist must determine the appropriate method for assessing brain death. According to current guidelines, how should brain death be evaluated in this patient?	It should not be determined in patients younger than 37 weeks gestational age.
	37	Case	A 55-year-old male patient is admitted to the intensive care unit following a severe traumatic brain injury. He is currently showing no signs of brainstem function other than spontaneous breathing, and remains in a deeply comatose state. The intensivist on call is considering the diagnosis of brain death and needs to determine the appropriate steps. According to current guidelines, how should the physician proceed with assessing brain death in this patient?	Brain death should not be assessed, as spontaneous breathing shows brainstem or brain function. The patient must not undergo brain death testing.
	38	Case	A 48-year-old female patient is being evaluated for brain death after suffering a massive intracerebral hemorrhage. During the assessment, it is noted that her core temperature is 34°C. What steps should the physician take regarding the patient's temperature to proceed with the brain death evaluation, according to current AAN guidelines?	According to current guidelines, the minimum core body temperature should be 36°C. For patients that had a lower temperature than 35.5°C previously it should be in place for more than 24h before initiating brain death assessment.
	39	Case	A 60-year-old male patient is in a deep coma following a catastrophic brain injury. The clinical team is considering the diagnosis of brain death. The initial clinical examination indicates the absence of all brainstem reflexes, but they wish to confirm this diagnosis with additional testing. The neurologist is evaluating the use of ancillary tests, such as EEGs, auditory evoked potentials (AEPs), or somatosensory evoked potentials (SEPs). According to current guidelines, how should these tests be utilized to assist with the diagnosis of brain death?	There is a clear recommendation against using these ancillary tests.

	40	Case	A 54-year-old male patient is in the ICU after a severe traumatic brain injury and is being evaluated for brain death. Clinical examination shows an absence of brainstem reflexes, and the attending physician considers using imaging as an ancillary test to confirm the diagnosis. The options include CT angiography, MRI angiography, or conventional 4-vessel catheter angiography. According to current guidelines, how should these imaging modalities be utilized to assist with the diagnosis of brain death?	Neither CT- nor MRI-angiography should be used. However, 4-vessel catheter angiography may be used according to the current guideline.
**Oral and topical treatment of painful diabetic polyneuropathy practice guideline update**	41	Knowledge	According to current guidelines, how and when should opioids be used in the treatment of diabetic polyneuropathy?	Clinicians should not use opioids for the treatment of painful diabetic neuropathy.
	42	Knowledge	According to current guidelines, should valproate/valproic acid be used in the treatment of diabetic polyneuropathy?	In all patients with painful diabetic neuropathy, clinicians should not prescribe valproic acid given the potential for serious adverse events unless multiple other effective medications have failed.
	43	Knowledge	According to current guidelines, what classes of oral medications have been shown to be effective in the treatment of diabetic polyneuropathy?	For patients with painful diabetic neuropathy, clinicians should consider offering tricyclic antidepressants, serotonin-norepinephrine reuptake inhibitors, gabapentinoids, and/or sodium channel blockers to alleviate pain.
	44	Knowledge	According to current guidelines, what should patients be counseled to be the goal of the pharmacological therapy of diabetic polyneuropathy?	"When initiating pharmacologic intervention for painful diabetic neuropathy, clinicians should counsel patients that the goal of therapy is to reduce, and not necessarily to eliminate, pain."
	45	Knowledge	According to current guidelines, what are options to offer patients with diabetic polyneuropathy except for oral therapies?	For patients who prefer topical, alternative, or nonpharmacologic treatments, healthcare providers may consider offering topical agents (such as capsaicin, glyceryl trinitrate spray, or Citrullus colocynthis), alternative therapies (such as ginkgo biloba), and/or nonpharmacologic interventions, including cognitive behavioral therapy, exercise, Tai Chi, or mindfulness practices.
	46	Case	A 62-year-old male with a history of type 2 diabetes presents to his primary care physician with progressively worsening pain in his feet and hands, consistent with diabetic polyneuropathy. He	Clinicians should not use opioids for the treatment of painful diabetic neuropathy.

			describes the pain as burning and difficult to tolerate, and first-line treatments, including gabapentin and lifestyle modifications, have provided limited relief. The physician considers whether opioids might be appropriate for pain management in this scenario. According to current guidelines, how and under what circumstances should opioids be utilized in the treatment of diabetic polyneuropathy?	
	47	Case	A 58-year-old female patient with painful diabetic neuropathy visits her neurologist for the first time seeking treatment options for her symptoms. She mentions that she has heard of valproic acid as a potential treatment and wonders if it could be a suitable option for her. Given that this is her initial inquiry into managing her neuropathy, what does current guidance suggest regarding the use of valproic acid as a first-line treatment?	In all patients with painful diabetic neuropathy, clinicians should not prescribe valproic acid given the potential for serious adverse events unless multiple other effective medications have failed.
	48	Case	A 60-year-old male patient with a history of type 2 diabetes presents to his primary care physician with complaints of persistent burning pain in his feet, consistent with diabetic polyneuropathy. He asks what treatment options are available to alleviate his pain. The physician considers current guidelines and effective classes of oral medications. What classes of medications should be considered for managing the patient's painful diabetic neuropathy?	For patients with painful diabetic neuropathy, clinicians should consider offering tricyclic antidepressants, serotonin-norepinephrine reuptake inhibitors, gabapentinoids, and/or sodium channel blockers to alleviate pain.
	49	Case	A 65-year-old female patient with painful diabetic neuropathy presents to her physician for initiation of pharmacological therapy. She expresses hope that the treatment will completely eliminate her pain. How should the physician counsel the patient regarding the realistic goals of pharmacological therapy for painful diabetic neuropathy, according to current guidelines?	"When initiating pharmacologic intervention for painful diabetic neuropathy, clinicians should counsel patients that the goal of therapy is to reduce, and not necessarily to eliminate, pain."
	50	Case	A 59-year-old male patient with painful diabetic polyneuropathy reports that he prefers to avoid oral medications and asks if there are other effective treatment options available. What options should the	For patients who prefer topical, alternative, or nonpharmacologic treatments, healthcare providers may consider offering topical agents (such as capsaicin, glyceryl trinitrate spray, or Citrullus

			physician offer to address the patient's symptoms, based on current guidelines for diabetic polyneuropathy?	colocynthis), alternative therapies (such as ginkgo biloba), and/or nonpharmacologic interventions, including cognitive behavioral therapy, exercise, Tai Chi, or mindfulness practices.
**Dopaminergic Therapy for Motor Symptoms in Early Parkinson Disease**	51	Knowledge	According to current guidelines, in patients with early Parkinson's Disease who seek treatment for motor symptoms, what should clinicians recommend as the initial preferential dopaminergic therapy?	For patients with early Parkinson's disease who wish to treat motor symptoms, clinicians should recommend levodopa as the preferred initial dopaminergic therapy. Dopamine-agonists may be precribed udner certain circmstances.
	52	Knowledge	According to current guidelines, are there any recommendations for age groups where the use of dopamine agonists is preferred over levodopa in the treatment of early Parkinson's Disease?	Below 60: May be prescribed for individuals with high risk of dyskinesiabut are not generally prefered. Above 70: Should be avoided due to higher risk of side effects.
	53	Knowledge	Are there any recommendations regarding comorbidities that would make the use of dopamine agonists contraindicated in the treatment of early Parkinson's Disease?	Clinicians should avoid prescribing dopamine agonists to patients with early-stage Parkinson's disease who are at greater risk for medication-related adverse effects. This includes individuals over 70 years of age, those with a history of impulse control disorders, and patients with pre-existing cognitive impairment, excessive daytime sleepiness, or hallucinations.
	54	Knowledge	According to current guidelines, what is the recommended approach regarding the initial dosing of dopamine agonists or levodopa for patients with early Parkinson's disease, particularly concerning dose escalation?	For patients with early Parkinson's disease, clinicians should use the lowest effective dose of levodopa - defined as the minimum dose that provides sufficient symptomatic relief - in order to reduce the risk of dyskinesia and other potential adverse effects.
	55	Knowledge	According to current guidelines, if dopamine agonists need to be discontinued in patients with early Parkinson's Disease, should the treatment be withdrawn or when possible gradually decreased?	Clinicians should taper or discontinue dopamine agonists if patients develop disabling side effects, such as impulse control disorders, excessive daytime sleepiness, sudden-onset sleep, cognitive issues, or hallucinations. If discontinuation is needed, clinicians should monitor for dopamine withdrawal syndrome and, when feasible, gradually reduce the dose to minimize symptoms.
	56	Case	A 60-year-old male patient recently diagnosed with early Parkinson's disease presents to his neurologist seeking treatment for his motor symptoms, which include tremors and bradykinesia. He asks about the	For patients with early Parkinson's disease who wish to treat motor symptoms, clinicians should recommend levodopa as the preferred initial

			best approach for managing these symptoms. What should the clinician recommend as the preferred initial dopaminergic therapy for motor symptoms in early Parkinson's disease, according to current guidelines?	dopaminergic therapy. Dopamine-agonists may be precribed under certain circmstances.
	57	Case	A 60-year-old male patient with early Parkinson's disease is discussing treatment options with his neurologist. He is curious about whether his age should influence the choice between starting with levodopa or a dopamine agonist. Are there any age-specific recommendations that would support the use of dopamine agonists over levodopa for treating early Parkinson's disease, according to current guidelines? Would they generally prefered in a certain age group?	Below 60: May be prescribed for individuals with high risk of dyskinesiabut are not generally prefered.<br>Above 70: Should be avoided due to higher risk of side effects.
	58	Case	A 62-year-old male patient has recently been diagnosed with early-stage Parkinson's disease. He also has a history of cognitive impairment and reports experiencing occasional hallucinations. During a discussion about treatment options, the neurologist is considering whether dopamine agonists might be an appropriate choice. What comorbidities or patient characteristics would make the use of dopamine agonists contraindicated in the treatment of early Parkinson's disease, based on current guidelines?	Clinicians should avoid prescribing dopamine agonists to patients with early-stage Parkinson's disease who are at greater risk for medication-related adverse effects. This includes individuals over 70 years of age, those with a history of impulse control disorders, and patients with pre-existing cognitive impairment, excessive daytime sleepiness, or hallucinations.
	59	Case	A 63-year-old male patient with early Parkinson's disease presents to his neurologist with concerns about his motor symptoms, including tremors and rigidity. The neurologist decides to initiate dopaminergic therapy to improve his quality of life. During the conversation, the patient expresses concern about potential side effects such as dyskinesia and asks how the medication will be managed to minimize these risks over time. What should the neurologist consider when determining the initial dose and adjusting the medication for this patient?	For patients with early Parkinson's disease, clinicians should use the lowest effective dose of levodopa - defined as the minimum dose that provides sufficient symptomatic relief - in order to reduce the risk of dyskinesia and other potential adverse effects.

	60	Case	A 64-year-old male patient with early Parkinson's disease has been on dopamine agonist therapy for over a year but has now developed impulse control issues and excessive daytime sleepiness. The neurologist decides to discontinue the dopamine agonist therapy. What is the appropriate approach for discontinuing the medication in this situation?	Clinicians should taper or discontinue dopamine agonists if patients develop disabling side effects, such as impulse control disorders, excessive daytime sleepiness, sudden-onset sleep, cognitive issues, or hallucinations. If discontinuation is needed, clinicians should monitor for dopamine withdrawal syndrome and, when feasible, gradually reduce the dose to minimize symptoms.
**Practice guideline update: Pharmacologic treatment for pediatric migraine prevention**	61	Knowledge	According to current guidelines, how well did treatments for the prophylaxis of migraine in children perform in clinical trials compared to placebo?	Most randomized controlled trials investigating the effectiveness of preventive medications for pediatric migraine have not shown a significant advantage over placebo. These trials indicate a high placebo response rate in pediatric migraine patients.
	62	Knowledge	According to current guidelines, are there any psychiatric comorbidities that are recommended to screen for when treating children with migraine?	Yes, children should be screened for mood and anxiety disorders.
	63	Knowledge	According to current guidelines, in case topiramate or valproic acid are considered for the prophylaxis of migraine in children, what are the recommended supplements in certain populations?	The guideline states: "Clinicians must recommend daily folic acid supplementation to patients of childbearing potential 6 who take topiramate or valproate"
	64	Knowledge	According to current guidelines, what medication that is used for the prophylaxis of migraine in children is known to decrease the efficacy of oral combined hormonal contraceptives?	Topiramate.
	65	Knowledge	According to current guidelines, which medication that is used for the prophylaxis of migraine in children is known to increase the risk of suicide?	Amitriptyline. Families should be councelled accordingly.
	66	Case	The parents of a 12-year-old girl bring her to a neurologist due to frequent and debilitating migraine headaches. They are concerned about her quality of life and ask about treatment options for preventing migraines. They have heard about preventive medications and wonder how effective these treatments are in children. After explaining the available medications, they ask whether these treatments have been found to perform significantly better than non-active interventions in clinical trials	Most randomized controlled trials investigating the effectiveness of preventive medications for pediatric migraine have not shown a significant advantage over placebo. These trials indicate a high placebo response rate in pediatric migraine patients.

			involving children. How should the neurologist address their concern based on current guidelines?	
	67	Case	The parents of a 10-year-old boy bring him to a pediatric neurologist due to frequent migraines that have begun affecting his school performance and social life. During the consultation, they mention that he often seems withdrawn and occasionally anxious. The neurologist considers how to best support the child beyond just treating his migraines. What additional screenings should the neurologist recommend according to current guidelines?	The neurologist should recommend screening for mood and anxiety disorders.
	68	Case	A 15-year-old female patient presents with chronic migraines that have been affecting her daily life, despite trials of several initial treatments. After discussing the options, the neurologist recommends topiramate for migraine prevention. The patient's mother expresses concern about making sure her daughter is well-supported while starting a new medication, particularly given her age and future health. How should the neurologist approach their concerns and ensure any additional needs are addressed according to current guidelines?	The guideline states: "Clinicians must recommend daily folic acid supplementation to patients of childbearing potential who take topiramate or valproate"
	69	Case	A 16-year-old female patient presents to her neurologist for the management of chronic migraines. The neurologist discusses potential preventive treatment options. During the conversation, the patient mentions that she uses oral combined hormonal contraceptives. The neurologist considers whether any of the suggested treatments might require extra attention or adjustment due to her contraceptive use. What should the neurologist take into consideration according to current guidelines when prescribing prophylactic medication in this situation?	The neurologist should take into consideration that topiramate, a medication used for the prophylaxis of migraines in children and adolescents, is known to decrease the efficacy of oral combined hormonal contraceptives.
	70	Case	A 14-year-old female patient presents with chronic migraines that significantly impact her school and social activities. After a thorough evaluation, her neurologist recommends starting a preventive	Amitriptyline. Families should be councelled accordingly.

			medication. During the discussion, the patient's mother expresses concern about any potential mental health risks associated with migraine treatments, given a family history of depression. The neurologist considers this information carefully while determining the best treatment strategy. What specific precautions and patient counseling should the neurologist undertake according to current guidelines, especially with regard to any increased risks related to mental health, according to current guidelines?	
**Practice guideline update: Acute treatment of migraine in children and adolescents**	71	Knowledge	According to current guidelines, what are recommended first-line treatment options for acute pain in adolescents with migraine?	Ibuprofen is commonly used and recommended. Certain triptans are also approved for use in adolescents and recommended in the guideline: sumatriptan, zolmitriptan, rizatriptan or almotriptan.
	72	Knowledge	According to current guidelines, in adolescents with migraine, should another triptane be offered if one class of triptans fails to provide proper pain relief?	Yes, clinicians should offer an alternate triptan.
	73	Knowledge	According to current guidelines, should a clinician counsel that for an adolescent suffering from migraine taking their triptane during a typical aura is safe or not?	Yes, it is recommended to counsel that it is safe but to point out that it may be more effective when taken at the onset of head pain.
	74	Knowledge	According to current guidelines, when should opioids be considered for migraine treatment in adolescents?	Opioids should not be considered for the treatment of migraine in adolescents.
	75	Knowledge	According to current guidelines and regarding medication overuse headache in adolescents with migraine, what would be the maximal recommended number of days per month for ibuprofen and acetaminophen?	Clinicians should advise patients and families to limit the use of acetaminophen or ibuprofen to no more than 14 days per month, and triptans to no more than 9 days per month. Additionally, the combined use of triptans, analgesics, or opioids should be restricted to no more than 9 days per month for over three consecutive months to prevent medication overuse headaches.
	76	Case	A 15-year-old adolescent presents to the clinic with frequent, severe migraines that significantly disrupt daily activities, including school and social engagements. The patient is seeking effective options for acute pain relief during migraine attacks. The physician is considering the best first-line	Ibuprofen is commonly used and recommended. Certain triptans are also approved for use in adolescents and recommended in the guideline: sumatriptan, zolmitriptan, rizatriptan or almotriptan.

			treatment options. During the discussion, the patient's mother mentions being hesitant about using strong medications, preferring options with a proven track record for adolescents. What should the physician recommend as appropriate treatment choices for acute migraine pain, following current guidelines?	
	77	Case	A 16-year-old adolescent patient with frequent migraines returns to her neurologist for follow-up. She was previously prescribed sumatriptan for acute migraine attacks, but reports that it hasn't provided adequate relief. The patient and her parents express frustration and are unsure about continuing with similar medications. The neurologist considers how to adjust her treatment plan based on the patient's lack of response to the initial therapy. What should the neurologist recommend regarding further options for migraine management, according to current guidelines?	Clinicians should offer an alternate triptan.
	78	Case	A 17-year-old adolescent experiencing migraines with typical aura asks whether it is safe to take her prescribed triptan during the aura phase instead of waiting for the headache to begin. How should the clinician address this question, based on current guidelines, while providing appropriate advice regarding timing and effectiveness?	Yes, it is recommended to counsel that it is safe but to point out that it may be more effective when taken at the onset of head pain.
	79	Case	A 16-year-old adolescent with chronic migraines, who has not found relief with several first-line treatments, presents to their neurologist. The parents, feeling increasingly desperate for a solution, ask whether opioids might be an option for pain relief. How should the neurologist respond, according to current guidelines?	Opioids should not be considered for the treatment of migraine in adolescents.
	80	Case	A 15-year-old adolescent with recurrent migraines visits her neurologist for a follow-up consultation. During the discussion, it becomes evident that she has been relying on ibuprofen and acetaminophen frequently to manage her migraine pain. The neurologist wants to ensure that her treatment plan	Clinicians should advise patients and families to limit the use of acetaminophen or ibuprofen to no more than 14 days per month, and triptans to no more than 9 days per month. Additionally, the combined use of triptans, analgesics, or opioids should be restricted to no more than 9 days per month for over

			remains both safe and effective. What guidance should the neurologist provide regarding the appropriate frequency of using these medications, according to current recommendations?	three consecutive months to prevent medication overuse headaches.
**Practice guideline update: Vaccine-preventable infections and immunization in multiple sclerosis**	81	Knowledge	According to recent guidelines, should patients with multiple sclerosis follow general local vaccination guidelines if no specific contraindications (such as immunomodulatory treatment) exist?	Yes, they should.
	82	Knowledge	According to current guidelines, are multiple sclerosis patients generally advised to get the yearly influenza vaccination?	"Clinicians should recommend that patients with MS receive the influenza vaccination annually, unless there is a specific contraindication."
	83	Knowledge	According to current guidelines, when and under which circumstances should vaccinations and immunizations be discussed with multiple sclerosis patients after diagnosis?	Clinicians should consider discussing the benefits of vaccination with patients soon after a diagnosis of multiple sclerosis, regardless of the initial treatment plan, to avoid future delays in starting immunosuppressive or immunomodulatory therapies. They will further need to be discussed and checked before initiating an immunomodulatory therapy.
	84	Knowledge	According to current guidelines, is it generally recommended to vaccinate multiple sclerosis patients during a relapse?	Clinicians should postpone vaccination for individuals with multiple sclerosis who are undergoing a relapse until the relapse has clinically resolved or is no longer active, such as when progression has halted but residual disability remains. This is typically several weeks after the onset of the relapse.
	85	Knowledge	According to current guidelines, are there recommendations regarding live attenuated vaccines in multiple sclerosis patients that are undergoing immunomodulatory treatment?	The guidelines generally advises against using live attenuated vaccines for individuals with multiple sclerosis receiving or recently stopping immunomodulatory therapies. However, in high-risk infection scenarios, such as pandemics or endemic areas, the potential benefits of live vaccines may outweigh the risks, particularly if killed vaccines are unavailable. In such cases, clinicians might consider recommending live vaccines
	86	Case	A 34-year-old female patient with multiple sclerosis asks her neurologist whether she needs any special considerations regarding vaccinations. What advice should the neurologist provide based on current guidelines?	a) Generally follow local guidelines; b) don't administer live-attenuated vaccines under immunotherapy, c) do not administer vaccines during relapse

	87	Case	A 45-year-old male patient with multiple sclerosis visits his neurologist during flu season and asks if he should get the yearly flu shot. What should the neurologist advise, considering current guidelines?	"Clinicians should recommend that patients with MS receive the influenza vaccination annually, unless there is a specific contraindication." CAVE: Live vaccine if patient under DMT.
	88	Case	A 38-year-old female patient is recently diagnosed with multiple sclerosis. During her consultation, she expresses that she does not plan to begin any treatment at this time. She asks whether her condition impacts her need for vaccinations or immunizations. When and under what circumstances should the neurologist discuss vaccinations with her, considering her decision and based on current guidelines?	Clinicians should consider discussing the benefits of vaccination with patients soon after a diagnosis of multiple sclerosis, regardless of the initial treatment plan, to avoid future delays in starting immunosuppressive or immunomodulatory therapies. They will further need to be discussed and checked before initiating an immunomodulatory therapy.
	89	Case	A 40-year-old male patient with multiple sclerosis is currently experiencing a relapse and is due for his scheduled vaccination. He asks if it is safe to proceed with the vaccination during his relapse. How should the neurologist respond based on current guidelines?	Clinicians should postpone vaccination for individuals with multiple sclerosis who are undergoing a relapse until the relapse has clinically resolved or is no longer active, such as when progression has halted but residual disability remains. This is typically several weeks after the onset of the relapse.
	90	Case	A 36-year-old patient with multiple sclerosis, currently undergoing therapy with dimethyl fumarate, is planning to travel to a region where yellow fever is endemic. They ask their neurologist whether they can receive the yellow fever vaccine before their trip. How should the neurologist approach this request?	The guidelines generally advises against using live attenuated vaccines for individuals with multiple sclerosis receiving or recently stopping immunomodulatory therapies. However, in high-risk infection scenarios, such as pandemics or endemic areas, the potential benefits of live vaccines may outweigh the risks, particularly if killed vaccines are unavailable. In such cases, clinicians might consider recommending live vaccines.
**Practice guideline: Treatment for insomnia and disrupted sleep behavior in children and adolescents with autism spectrum disorder**	91	Knowledge	According to current guidelines, what should be the first-line treatment approach for treating sleep disorders in children or adolescents with autism spectrum disorder?	Behavioral strategies such as cognitive behavioral therapy should be used as first-line therapy.
	92	Knowledge	According to current guidelines, if behavioral treatment strategies have been unsuccessful for the treatment of sleep disorders in children or adolescents with autism spectrum disorder, what should be the next treatment approach?	When managing sleep difficulties in children and adolescents with ASD, pharmacologic strategies like melatonin can be considered if behavioral approaches fail. Melatonin shows low to moderate effectiveness, particularly for sleep onset and

				maintenance, with prescription-grade being preferred over OTC due to dosage variability.
	93	Knowledge	According to current guidelines, what should be an initial dose of melatonin for the treatment of sleep disorders in children or adolescents with autism spectrum disorder?	When prescribing melatonin for sleep issues in children and adolescents with ASD, clinicians should begin with a low dose (1–3 mg per day), administered 30–60 minutes before bedtime, and adjust gradually as needed, with a maximum dose of 10 mg per day.
	94	Knowledge	According to current guidelines, is there any evidence for the use of weighted blankets in the treatment of sleep disorders in children or adolescents with autism spectrum disorder?	Clinicians should inform children and adolescents with ASD experiencing sleep disturbances, along with their parents, that there is currently no evidence supporting the regular use of weighted blankets or specialized mattress technology to improve disrupted sleep.
	95	Knowledge	According to current guidelines, what should clinicians counsel children or adolescents with autism spectrum disorder and their families about the longterm safety data for melatonin for the treatment of sleep disorders?	When prescribing melatonin for sleep disturbances in children and adolescents with ASD, clinicians should inform both the patients (as appropriate) and their parents about the possible adverse effects of melatonin use and the absence of long-term safety data.
	96	Case	The parents of a 12-year-old boy with autism spectrum disorder bring him to his pediatrician, concerned about his ongoing sleep problems. They ask about the best approach to help him sleep better. What should the pediatrician recommend as the initial treatment strategy, according to current guidelines?	Behavioral strategies such as cognitive behavioral therapy should be used as first-line therapy.
	97	Case	A 10-year-old child with autism spectrum disorder is brought to the clinic by his parents, who report that despite trying various behavioral strategies, his sleep difficulties persist. They are seeking further options to help their child sleep better. What treatment approach should the physician consider next, according to current guidelines?	When managing sleep difficulties in children and adolescents with ASD, pharmacologic strategies like melatonin can be considered if behavioral approaches fail. Melatonin shows low to moderate effectiveness, particularly for sleep onset and maintenance, with prescription-grade being preferred over OTC due to dosage variability.
	98	Case	The parents of an 11-year-old child with autism spectrum disorder consult their pediatrician regarding their child's ongoing sleep difficulties. After unsuccessful attempts with behavioral strategies, the physician suggests melatonin might be an option. The parents ask how they should begin administering	When prescribing melatonin for sleep issues in children and adolescents with ASD, clinicians should begin with a low dose (1–3 mg per day), administered 30–60 minutes before bedtime, and adjust gradually as needed, with a maximum dose of 10 mg per day.

			it and how much would be appropriate at first. What guidance should the physician provide to the parents, considering current best practices for melatonin use?	
	99	Case	The parents of a 9-year-old child with autism spectrum disorder bring him to his pediatrician, concerned about his ongoing sleep issues. They mention that they are considering using a weighted blanket, having heard that it may help him sleep better. What should the pediatrician advise regarding the use of weighted blankets to address sleep disturbances, based on current guidelines?	Clinicians should inform children and adolescents with ASD experiencing sleep disturbances, along with their parents, that there is currently no evidence supporting the regular use of weighted blankets or specialized mattress technology to improve disrupted sleep.
	100	Case	The parents of a 13-year-old adolescent with autism spectrum disorder are discussing treatment options for their child's sleep disturbances with the pediatrician. The pediatrician suggests melatonin as a possible option, but the parents express concerns about the safety of using it over a long period. What should the pediatrician counsel the parents regarding melatonin's long-term safety profile and potential side effects, according to current guidelines?	When prescribing melatonin for sleep disturbances in children and adolescents with ASD, clinicians should inform both the patients (as appropriate) and their parents about the possible adverse effects of melatonin use and the absence of long-term safety data.
**Practice advisory update: Patent foramen ovale and secondary stroke prevention**	101	Knowledge	According to current guidelines, in patients with stroke and a foramen ovale, is there a need to rule out other reasons of stroke before considering closing it?	For patients being considered for patent foramen ovale (PFO) closure, clinicians should confirm that a comprehensive evaluation has been completed to exclude other potential causes of stroke, consistent with the protocols used in all successful PFO closure trials.
	102	Knowledge	According to current guidelines, is there a clear recommendation for patients with a stroke, a foramen ovale and thrombophilia regarding the closure of the foramen ovale?	For patients who are otherwise suitable candidates for PFO closure but need long-term anticoagulation due to suspected or confirmed hypercoagulability (such as diagnosed thrombophilia, unprovoked deep vein thrombosis, or unprovoked pulmonary embolism), clinicians should advise that the effectiveness of PFO closure in conjunction with anticoagulation remains uncertain.
	103	Knowledge	According to current guidelines, is a baseline ECG strictly necessary for patients with a stroke and a foramen ovale before considering closure?	Yes, it is definitely recommended, especially to screen for atrial fibrillation.

	104	Knowledge	According to current guidelines, do patients with a stroke and a foramen ovale, how should the shunt size be evaluated?	For patients being evaluated for PFO closure, clinicians should first use transthoracic echocardiography (TTE) to assess potential cardioembolic sources, followed by transesophageal echocardiography (TEE) if TTE does not reveal a high-risk stroke mechanism. Bubble contrast should be used in these studies, both with and without the Valsalva maneuver, to identify right-to-left shunts and evaluate the extent of shunting.
	105	Knowledge	According to current guidelines, in patients with a stroke and a foramen ovale, what are the age groups a closure is typically recommended for given this is the most likely cause of the stroke?	Typically in patients younger than 60 if no other mechanism is identified. The guideline further mentions that it may be offered in other populations (such as 60-65) year olds with very limited other risk factors.
	106	Case	A 55-year-old male patient who recently experienced a stroke is found to have a patent foramen ovale (PFO) on imaging. He is referred to a specialist to discuss the possibility of PFO closure. Before proceeding, what steps should the clinician take to ensure appropriate management in line with current guidelines?	For patients being considered for patent foramen ovale (PFO) closure, clinicians should confirm that a comprehensive evaluation has been completed to exclude other potential causes of stroke, consistent with the protocols used in all successful PFO closure trials.
	107	Case	A 47-year-old female patient presents to her neurologist for follow-up after experiencing an ischemic stroke three months ago. During her evaluation, a patent foramen ovale (PFO) was discovered. The work-up included an MRI, which confirmed the ischemic stroke in the right parietal lobe. Additionally, several other findings emerged, including a normal carotid Doppler ultrasound, no atrial fibrillation on extended cardiac monitoring, and no evidence of large vessel disease. The MRI also revealed only minor, age-related white matter changes. Laboratory testing confirmed an underlying thrombophilia, and the patient has a history of an unprovoked deep vein thrombosis. She currently started long-term anticoagulation therapy. The patient also has mildly elevated liver enzymes, an unrelated incidental finding that her primary care physician is monitoring.	For patients who are otherwise suitable candidates for PFO closure but need long-term anticoagulation due to suspected or confirmed hypercoagulability (such as diagnosed thrombophilia, unprovoked deep vein thrombosis, or unprovoked pulmonary embolism), clinicians should advise that the effectiveness of PFO closure in conjunction with anticoagulation remains uncertain.

			During the consultation, the patient inquires whether closing the PFO might reduce her risk of future strokes, considering all her findings. How should the neurologist reason through these findings and weigh the decision regarding PFO closure, based on current guidelines?	
	108	Case	A 55-year-old male patient presents for a follow-up after an ischemic stroke. Further screening confirmed a patent foramen ovale (PFO), and the neurologist is considering closure. The patient has undergone multiple evaluations, but a baseline ECG has not yet been performed. He is eager to proceed with PFO closure. What is the importance of obtaining an ECG in this scenario, according to current guidelines?	Yes, it is definitely recommended, especially to screen for atrial fibrillation.
	109	Case	A 50-year-old male patient presents for evaluation after an ischemic stroke. A patent foramen ovale (PFO) was identified during the initial work-up. A transthoracic echocardiogram (TTE) was performed but did not fully assess the characteristics of the shunt. The neurologist is now considering further imaging to decide on the suitability of PFO closure. What steps should be taken to evaluate the shunt size and characteristics more comprehensively, according to current guidelines?	For patients being evaluated for PFO closure, clinicians should first use transthoracic echocardiography (TTE) to assess potential cardioembolic sources, followed by transesophageal echocardiography (TEE) if TTE does not reveal a high-risk stroke mechanism. Bubble contrast should be used in these studies, both with and without the Valsalva maneuver, to identify right-to-left shunts and evaluate the extent of shunting.
	110	Case	A 57-year-old patient presents for follow-up after a recent ischemic stroke, during which a patent foramen ovale (PFO) was identified. No other stroke mechanism has been determined, and the patient is interested in exploring PFO closure. Given the patient's age, and considering whether the closure might also be appropriate for other age groups, how should the neurologist proceed with recommendations based on current guidelines?	According to current guidelines, PFO closure is typically recommended for patients under 60 years of age when no other mechanism for the stroke has been identified, and the PFO is believed to be the likely cause. In the case of patients between 60 and 65 years of age, PFO closure may still be considered if other stroke risk factors are minimal or absent, but this is less clear-cut than for younger patients. Given the patient's age of 57, closure could be recommended, provided that no other stroke mechanisms are evident.
**Stroke Prevention in Symptomatic Large**	111	Knowledge	According to current guidelines, is anticoagulation using for example warfarin a good option for patients	No, according to the guideline it has not shown superiority over ASS while having a worse risk profile

**Artery Intracranial Atherosclerosis Practice Advisory**			with symptomatic intracranial atherosclerotic arterial stenosis?	and is thus not recommended. Aspirin is the typical recommendation, depending on the exact situation and time combined with other drugs (e.g. clopidogrel).
	112	Knowledge	According to current guidelines, which patients with a history of symptomatic intracranial atherosclerotic arterial stenosis should clopidogrel and cilostazol be added to aspirin?	Clinicians should consider adding clopidogrel (75 mg per day) to aspirin for up to 90 days to reduce stroke risk in patients with severe symptomatic intracranial atherosclerosis (70%-99% stenosis) who have a low risk of hemorrhagic transformation. Alternatively, cilostazol (200 mg per day) may be added to aspirin under similar conditions, particularly in Asian patients or as an alternative to clopidogrel.
	113	Knowledge	According to current guidelines, what is the LDL goal in patients with symptomatic intracranial atherosclerotic arterial stenosis?	LDL <70 mg/dL.
	114	Knowledge	According to current guidelines, what is the longterm blood pressure target in patients with symptomatic intracranial atherosclerotic arterial stenosis?	Clinicians should recommend a long-term blood pressure target of <140/90 mm Hg.
	115	Knowledge	According to current guidelines, should a direct bypass treatment be recommended to patients with symptomatic intracranial atherosclerotic arterial stenosis?	Clinicians should not recommend direct bypass for stroke prevention in patients with s-ICAS.
	116	Case	A 63-year-old male patient presents with symptomatic intracranial arterial stenosis confirmed on imaging. The neurologist is considering treatment options to prevent recurrent ischemic events. The patient has heard about warfarin as an anticoagulant therapy and wonders if it could be an effective option for his condition. How should the neurologist address the patient's inquiry, and what does current guidance suggest about the role of anticoagulation compared to antiplatelet therapy in managing intracranial atherosclerotic stenosis?	No, according to the guideline it has not shown superiority over ASS while having a worse risk profile and is thus not recommended. Aspirin is the typical recommendation, depending on the exact situation and time combined with other drugs (e.g. clopidogrel).
	117	Case	A 68-year-old male patient with a history of symptomatic intracranial arterial stenosis presents for follow-up. Imaging reveals a 75% stenosis in the middle cerebral artery, and he is currently taking	Clinicians should consider adding clopidogrel (75 mg per day) to aspirin for up to 90 days to reduce stroke risk in patients with severe symptomatic intracranial

			aspirin as part of his stroke prevention strategy. The neurologist is considering whether additional treatment should be introduced to better manage his condition and reduce the risk of further events. How should the neurologist approach this decision, considering the patient's current regimen, the severity of the stenosis, and current guidelines?	atherosclerosis (70%-99% stenosis) who have a low risk of hemorrhagic transformation. Alternatively, cilostazol (200 mg per day) may be added to aspirin under similar conditions, particularly in Asian patients or as an alternative to clopidogrel.
	118	Case	A 65-year-old female patient presents with symptomatic intracranial arterial stenosis and wants to understand the goals of her treatment plan, particularly with respect to cholesterol management. During the discussion, she asks what her target cholesterol levels should be to help reduce her risk of future strokes. How should the neurologist guide her, in line with current guidelines?	LDL <70 mg/dL.
	119	Case	A 62-year-old female patient with symptomatic intracranial atherosclerotic stenosis visits her neurologist to discuss her ongoing management plan. She is interested in understanding how her blood pressure should be controlled to reduce her risk of future strokes. What should the neurologist recommend as a target blood pressure for this patient, according to current guidelines?	Clinicians should recommend a long-term blood pressure target of <140/90 mm Hg.
	120	Case	A 59-year-old male patient with symptomatic intracranial arterial stenosis returns for follow-up. He has been reading about different interventions that could potentially improve outcomes in patients with narrowed vessels in the brain. During the consultation, he inquires about whether there are any surgical options that could enhance blood flow and prevent further events. How should the neurologist approach the patient's questions, considering current recommendations and treatment strategies?	Clinicians should not recommend direct bypass for stroke prevention in patients with s-ICAS.
**Practice Guideline: The treatment of tics in people with Tourette syndrome**	121	Knowledge	According to current guidelines, is watch and wait a recommended approach for children with tic disorders not suffering from functional impairment?	Yes, and clinicians should inform patients and caregivers that it is (as long as they do not experience functional impairment due to the tics).
	122	Knowledge	According to current guidelines, what - except for watch and wait - are the first-line treatment options	Clinicians may prescribe Comprehensive Behavioral Intervention for Tics (CBIT) in this case.

**and chronic tic disorders**			for children with tic disorders without functional impairment?	
	123	Knowledge	According to current guidelines, are botulinum toxin injections recommended for the treatment of tics in some children?	Physicians may consider botulinum toxin injections for older adolescents and adults with localized and bothersome motor tics or with severely disabling vocal tics, if the expected benefits outweigh the risks. Patients should be informed that the treatment may lead to temporary side effects, such as muscle weakness or reduced vocal strength.
	124	Knowledge	According to current guidelines, what is the recommended pharmaceutical treatment for children with tics and comorbid ADHD?	The guideline states: "Physicians should counsel individuals with tics and comorbid ADHD that alpha-2 adrenergic agonists may provide therapeutic benefit for both conditions".
	125	Knowledge	According to current guidelines, what are prerequisites for considering deep brain stimulation for the treatment of resistant tics in Tourette Syndrome?	Before prescribing DBS for medication-resistant motor and vocal tics in patients with Tourette Syndrome (TS), multiple classes of medications, including antipsychotics, dopamine depleters, and alpha-2 agonists, must have been administered, and CBIT must have been attempted or determined to be contraindicated. Further, a multidisciplinary evaluation involving a psychiatrist or neurologist, a neurosurgeon, and a neuropsychologist must be conducted to assess whether the potential benefits of DBS outweigh the associated risks. Physicians should confirm the DSM-5 diagnosis of Tourette Syndrome and exclude secondary and functional tic-like movements before considering DBS. A mental health professional must evaluate patients preoperatively for any psychiatric conditions that could affect the long-term success of the therapy and provide ongoing postoperative support. DBS may be considered for individuals with severe, self-injurious tics, such as severe cervical tics that risk causing spinal injury.
	126	Case	The parents of an 8-year-old boy bring him to a pediatric neurologist after noticing the onset of tics over the past few months. The child seems largely unaffected by the tics and continues to perform well	The neurologist should recommend a "watch and wait" approach since the child is not experiencing any functional impairment due to the tics. According to current guidelines, if the tics are not causing

			in school and social activities without any significant issues. The parents are worried but unsure if any treatment is necessary at this stage. What should the neurologist recommend regarding management, considering the current guidelines?	significant difficulties in daily life or affecting school or social activities, active intervention is typically not required. The neurologist should also inform the parents that this approach is appropriate and can be re-evaluated if the tics begin to interfere with the child's well-being.
	127	Case	The parents of a 10-year-old boy with tic disorders bring him to see a pediatric neurologist. The child is managing well at school and during social activities, but the parents are still concerned about his tics and want to know if there are any treatment options available that could help reduce them, even if they are not causing any major issues at present. What non-pharmacological options might the neurologist consider offering, according to current guidelines?	Clinicians may prescribe Comprehensive Behavioral Intervention for Tics (CBIT) in this case.
	128	Case	The parents of a 16-year-old adolescent with persistent, bothersome motor tics visit the neurologist for further guidance. The tics, although not severely disabling, have localized and are affecting his daily comfort. Despite trying behavioral therapy and medication with limited success, they wonder if there are other treatment options that could help specifically with these bothersome localized movements. Given current guidelines, how should the neurologist approach this inquiry and what treatment considerations might be discussed?	Physicians may consider botulinum toxin injections for older adolescents and adults with localized and bothersome motor tics or with severely disabling vocal tics, if the expected benefits outweigh the risks. Patients should be informed that the treatment may lead to temporary side effects, such as muscle weakness or reduced vocal strength.
	129	Case	The parents of a 10-year-old boy with tic disorder and ADHD come to the clinic seeking advice on managing both conditions. The child's symptoms are causing issues at school, particularly with attention and behavior, in addition to his tics. They want to know if there are any treatment options that might help with both problems simultaneously. Based on current guidelines, what approach should the neurologist suggest?	The guideline states: "Physicians should counsel individuals with tics and comorbid ADHD that alpha-2 adrenergic agonists may provide therapeutic benefit for both conditions".
	130	Case	A 22-year-old male patient with a long-standing diagnosis of Tourette Syndrome presents with severe, treatment-resistant motor and vocal tics.	Before prescribing DBS for medication-resistant motor and vocal tics in patients with Tourette Syndrome (TS), multiple classes of medications,

			Despite trying multiple medications, including antipsychotics, dopamine depleters, and other agents, as well as attempting behavioral therapy, his symptoms remain debilitating—particularly due to severe cervical tics that are self-injurious. The patient and his family are desperate to explore further interventions to improve his quality of life.<br>Could intracranial stimulation be a viable option, and what steps should be taken to evaluate its appropriateness for this patient?	including antipsychotics, dopamine depleters, and alpha-2 agonists, must have been administered, and CBIT must have been attempted or determined to be contraindicated. Further, a multidisciplinary evaluation involving a psychiatrist or neurologist, a neurosurgeon, and a neuropsychologist must be conducted to assess whether the potential benefits of DBS outweigh the associated risks. Physicians should confirm the DSM-5 diagnosis of Tourette Syndrome and exclude secondary and functional tic-like movements before considering DBS. A mental health professional must evaluate patients preoperatively for any psychiatric conditions that could affect the long-term success of the therapy and provide ongoing postoperative support. DBS may be considered for individuals with severe, self-injurious tics, such as severe cervical tics that risk causing spinal injury.

Supplementary Table 2: Included Guidelines Published by the American Academy of Neurology

Name	Date Published	Date Reaffirmed
Teratogenesis, Perinatal, and Neurodevelopmental Outcomes After In Utero Exposure to Antiseizure Medication	May 15, 2024	N/A
Pediatric and Adult Brain Death/Death by Neurologic Criteria Consensus Practice Guideline	December 6, 2023	N/A
Stroke Prevention in Symptomatic Large Artery Intracranial Atherosclerosis	March 21, 2022	N/A
Oral and topical treatment of painful diabetic polyneuropathy practice guideline update	December 27, 2021	N/A
Antiseizure medication withdrawal in seizure-free patients practice advisory update	December 6, 2021	N/A
Dopaminergic Therapy for Motor Symptoms in Early Parkinson Disease	November 15, 2021	N/A
Guidelines for the Prevention, Diagnosis, and Treatment of Lyme Disease	November 30, 2020	October 21, 2023
Patent foramen ovale and secondary stroke prevention	April 29, 2020	July 14, 2023
Treatment for Insomnia and Disrupted Sleep Behavior in Children and Adolescents with Autism Spectrum Disorder	February 12, 2020	February 25, 2023
Vaccine-preventable Infections and Immunization in Multiple Sclerosis	August 28, 2019	October 22, 2022
Acute Treatment of Migraine in Children and Adolescents	August 14, 2019	October 22, 2022
Pharmacologic Treatment for Pediatric Migraine Prevention	August 14, 2019	October 22, 2022
Treatment of Tics in People with Tourette Syndrome and Chronic Tic Disorders	May 6, 2019	April 30, 2022

Supplementary Table 3: Large Language Models and Respective Settings

Model	Exact Version	Temperature	Top_p	Other Settings	Approximate Training Data Cut-off
**GPT-4**	gpt-4-turbo-2024-04-09	0.2	1 (Default)	Default	December 2023
**GPT-4o (with and without RAG)**	gpt-4o-2024-11-20	0.2	1 (Default)	Default	October 2023 (CAVE: RAG version with access to guideline documents)
**GPT-4o mini**	gpt-4o-mini-2024-07-18	0.2	1 (Default)	Default	October 2021
**Gemini-1.5 Pro**	gemini-1.5-pro-002	0.2	0.95 (Default)	Safety settings (HARM_CATEGORY_HATE_SPEECH, HARM CATEGORY DANGEROUS CONTENT, HARM CATEGORY SEXUALLY EXPLICIT, HARM CATEGORY HARASSMENT) all set to BLOCK_NONE	September 2024
**LLaMA3-70b**	llama3-70b-8192	0.2	1 (Default)	Default	March 2023
**Mixtral-8x7b**	mixtral-8x7b-32768	0.2	1 (Default)	Default	Late 2023
**LLaMA3.1-Nemotron-70b-instruct**	llama-3.1-nemotron-70b-instruct	0.2	1 (Default)	Default	December 2023
**LLaMA3.1-Sonar-405k-Perplexity (with online search)**	llama-3.1-sonar-huge-128k-online	0.2 (Default)	0.9 (Default)	Default	December 2023 (CAVE: Live Access to internet sources)

Supplementary Table 4: Post-Hoc Bonferroni-Adjusted Wilcoxon Test Results for Comparing Language Model Ratings

Original Friedman test was also statistically significant, with χ^2 (8) = 228.7; P<.0001.

Model 1	Model 2	P-Value Corrected	Significantly Different
GPT-4o + RAG	LLaMA3.1-Sonar-405b + Online	P=0.005	Yes
GPT-4o + RAG	GPT-4o	P<.001	Yes
GPT-4o + RAG	GPT-4 Turbo	P<.001	Yes
GPT-4o + RAG	GPT-4o mini	P<.001	Yes
GPT-4o + RAG	LLaMA3.1-Nemotron-70b	P<.001	Yes
GPT-4o + RAG	LLaMA3-70b	P<.001	Yes
GPT-4o + RAG	Gemini-1.5-Pro	P<.001	Yes
GPT-4o + RAG	Mixtral-8x7b	P<.001	Yes
LLaMA3.1-Sonar-405b + Online	GPT-4o	P>.99	No
LLaMA3.1-Sonar-405b + Online	GPT-4 Turbo	P=0.008	Yes
LLaMA3.1-Sonar-405b + Online	GPT-4o mini	P=0.001	Yes
LLaMA3.1-Sonar-405b + Online	LLaMA3.1-Nemotron-70b	P<.001	Yes
LLaMA3.1-Sonar-405b + Online	LLaMA3-70b	P<.001	Yes
LLaMA3.1-Sonar-405b + Online	Gemini-1.5-Pro	P<.001	Yes
LLaMA3.1-Sonar-405b + Online	Mixtral-8x7b	P<.001	Yes
GPT-4o	GPT-4 Turbo	P=0.004	Yes
GPT-4o	GPT-4o mini	P<.001	Yes
GPT-4o	LLaMA3.1-Nemotron-70b	P<.001	Yes
GPT-4o	LLaMA3-70b	P<.001	Yes
GPT-4o	Gemini-1.5-Pro	P<.001	Yes
GPT-4o	Mixtral-8x7b	P<.001	Yes
GPT-4 Turbo	GPT-4o mini	P>.99	No
GPT-4 Turbo	LLaMA3.1-Nemotron-70b	P=0.52	No
GPT-4 Turbo	LLaMA3-70b	P>.99	No
GPT-4 Turbo	Gemini-1.5-Pro	P=0.002	Yes
GPT-4 Turbo	Mixtral-8x7b	P<.001	Yes
GPT-4o mini	LLaMA3.1-Nemotron-70b	P>.99	No
GPT-4o mini	LLaMA3-70b	P>.99	No
GPT-4o mini	Gemini-1.5-Pro	P=0.09	No
GPT-4o mini	Mixtral-8x7b	P=0.001	Yes
LLaMA3.1-Nemotron-70b	LLaMA3-70b	P>.99	No
LLaMA3.1-Nemotron-70b	Gemini-1.5-Pro	P=0.91	No
LLaMA3.1-Nemotron-70b	Mixtral-8x7b	P=0.11	No
LLaMA3-70b	Gemini-1.5-Pro	P=0.25	No
LLaMA3-70b	Mixtral-8x7b	P=0.05	Yes
Gemini-1.5-Pro	Mixtral-8x7b	P>.99	No

Supplementary Table 5: Cosine Similarity and BLEU Score Metrics for Language Models (Model Responses to Sample Answer)

Model	Cosine Similarity (Median)	Cosine Similarity (IQR)	BLEU Score (Median)	BLEU Score (IQR)
GPT-4o + RAG	0.3472	0.2159	0.0046	0.0082
GPT-4o	0.3208	0.2362	0.0043	0.0087
LLaMA3.1-Sonar-405b + Online	0.3170	0.2384	0.0075	0.0163
GPT-4o mini	0.2974	0.2193	0.0056	0.0096
GPT-4 Turbo	0.2933	0.2061	0.0048	0.0105
LLaMA3-70b	0.2458	0.2038	0.0048	0.0102
Gemini-1.5-Pro	0.2317	0.1931	0.0042	0.0058
LLaMA3.1-Nemotron-70b	0.2286	0.1942	0.0022	0.0028
Mixtral-8x7b	0.2048	0.1922	0.0053	0.0120

Supplementary Table 6: Ordinal Logistic Regression Results by Model Comparing Performance in Case-Based and Knowledge-Based Questions

Note: Odds Ratios represent the likelihood of higher performance ('Correct' > 'Inaccurate' > 'Wrong') for 'Knowledge' relative to 'Case'.

Model	P-value (Uncorrected)	P-value (Corrected)	Significant?	Odds Ratio	95% CI Lower	95% CI Upper
Gemini-1.5-Pro	P=0.04	P=0.38	No	0.7206	0.5249	0.9893
GPT-4o	P=0.44	P>.99	No	0.8751	0.6214	1.232
GPT-4o + RAG	P=0.001	P=0.01	Yes	2.42	1.415	4.138
GPT-4o mini	P=0.90	P>.99	No	0.9795	0.7119	1.348
GPT-4 Turbo	P=0.52	P>.99	No	1.113	0.806	1.537
LLaMA3.1-Nemotron-70b	P=0.22	P>.99	No	1.22	0.888	1.677
LLaMA3.1-Sonar-405b + Online	P=0.01	P=0.13	No	1.574	1.096	2.262
LLaMA3-70b	P=0.72	P>.99	No	1.06	0.77	1.459
Mixtral-8x7b	P<.001	P<.001	Yes	0.5191	0.3759	0.7167

Supplementary Figure 1: Heatmap of Post-Hoc Bonferroni-Adjusted Wilcoxon Test Results for Language Model Ratings

Heatmap showing pairwise Bonferroni-adjusted p-values for comparing language model ratings. The original Friedman test was statistically significant ($\chi^2(8) = 228.7$; $P<.0001$). Significance thresholds are indicated by a color gradient, with diagonal and upper triangle entries masked. See Supplementary Table 4 for underlying values.

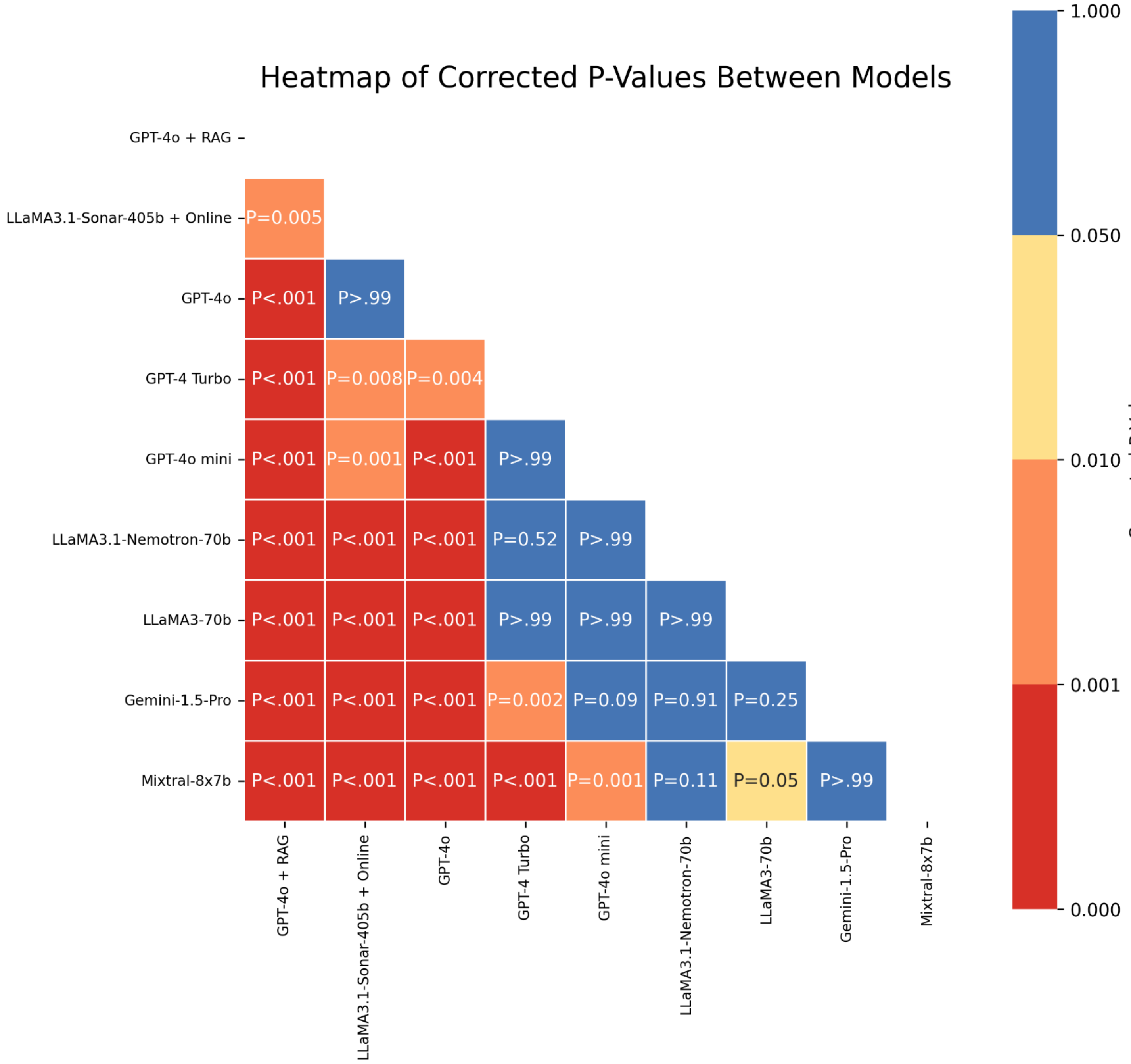

Supplementary Figure 2: Quality and Source Referencing of LLM Responses for Cases and Knowledge Questions

(a) Stacked bar chart illustrating the percentage of answers in each rating category—“Correct” (blue), “Inaccurate” (yellow), and “Wrong” (red)—when models responded to clinically oriented “Cases” questions.

(b) Corresponding stacked bar chart displaying the same rating categories for “Knowledge” questions. Despite asking for the same information, the “Cases” questions were phrased in a more clinical, scenario-based manner; retrieval-augmented generation (“RAG” or “Online”) approaches generally showed greater difficulty with this style.

(c) Stacked bar chart showing the distribution of source references for “Cases,” categorized as “Existing Sources” (dark green), “General Claim” (light green), “Source Hallucination” (yellow), and their variations with RAG or online augmentation (hatched).

(d) Source-referencing patterns for “Knowledge” questions following the same categorization.

All models were asked a set of “Cases” questions and “Knowledge” questions (N=65 for each domain) four times, yielding 520 total answers per domain per model. Due to rounding, summed percentages may not equal exactly 100%. Results of an ordinal logistic regression comparing model performance model in both question types can be found in Supplementary Table 6.

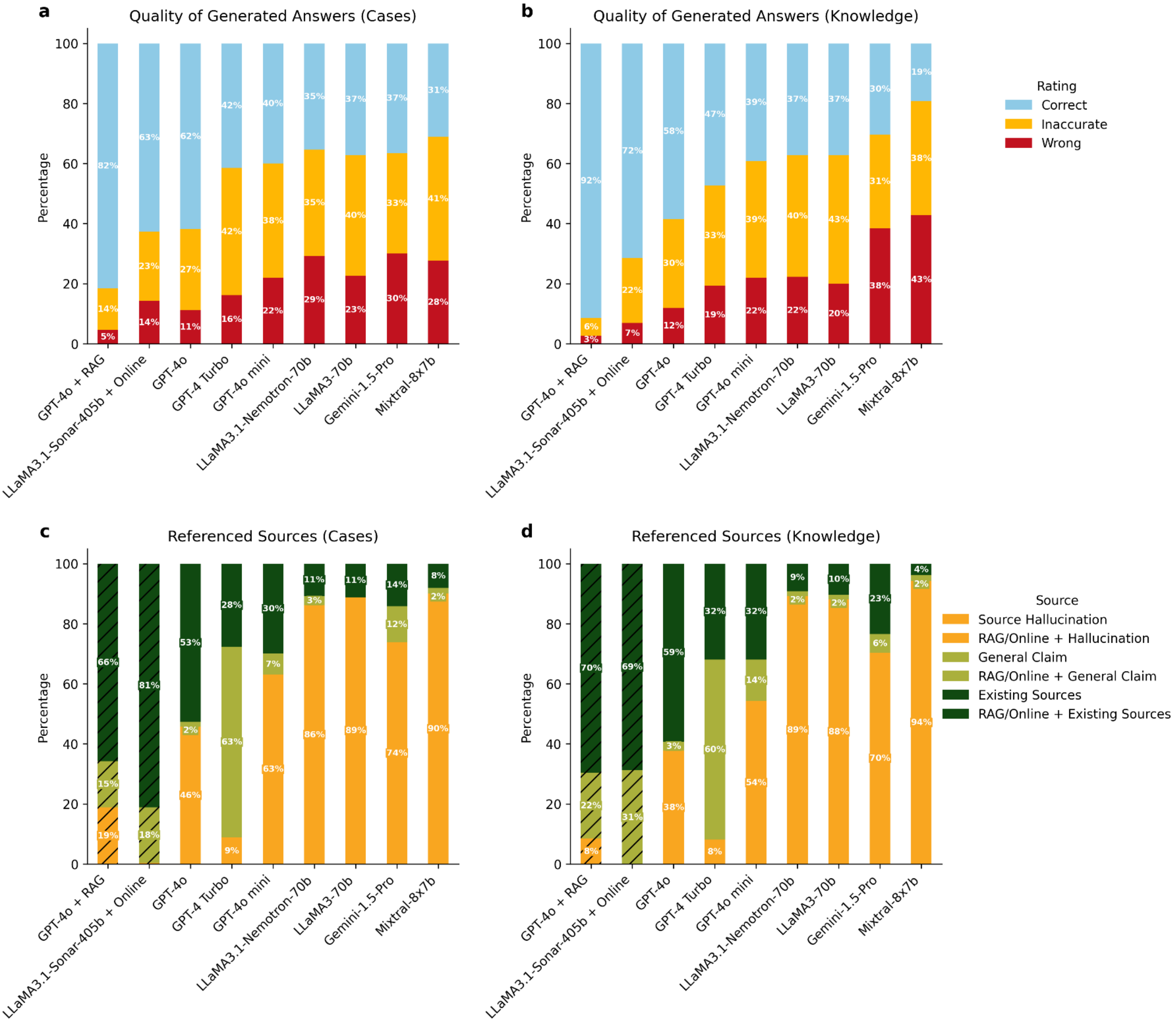

Supplementary Figure 3: Percentage of Correct Ratings per Guideline (Year) and Model

Line plot showing the percentage of responses evaluated as “Correct” (fully aligned with AAN guidelines) for each model across multiple guidelines, organized by publication year. The x-axis labels the guidelines and corresponding years, while the y-axis indicates the proportion of model-generated answers rated “Correct.” Each line (colored as per the legend) represents a distinct LLM setup, illustrating performance variability by clinical topic and model. AAN: American Academy of Neurology; ASM: Anti-seizure medication; ICAS: Intracranial Atherosclerotic Stenosis; LLM: Large Language Model; MS: Multiple Sclerosis; PFO: Patent Foramen Ovale; RAG: Retrieval-Augmented Generation; Tx: Treatment

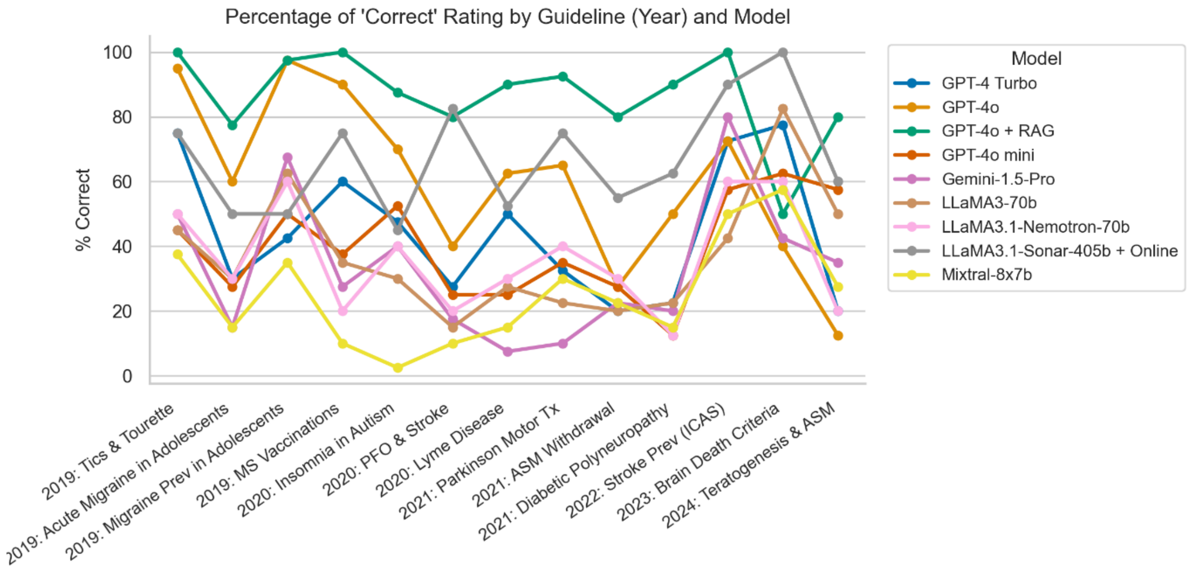

Supplementary Figure 4: Percentage of “Source Hallucination” by Guideline (Year) and Model

Line chart illustrating the percentage of responses that contained “Source Hallucination” (i.e., citing non-existent or incorrect references). The x-axis denotes various guidelines (by year), and the y-axis indicates how frequently each model’s answers included hallucinated sources. Each line (colored as per legend) corresponds to a distinct LLM or RAG-based system. AAN: American Academy of Neurology; ASM: Anti-seizure medication; ICAS: Intracranial Atherosclerotic Stenosis; LLM: Large Language Model; MS: Multiple Sclerosis; PFO: Patent Foramen Ovale; RAG: Retrieval-Augmented Generation; Tx: Treatment

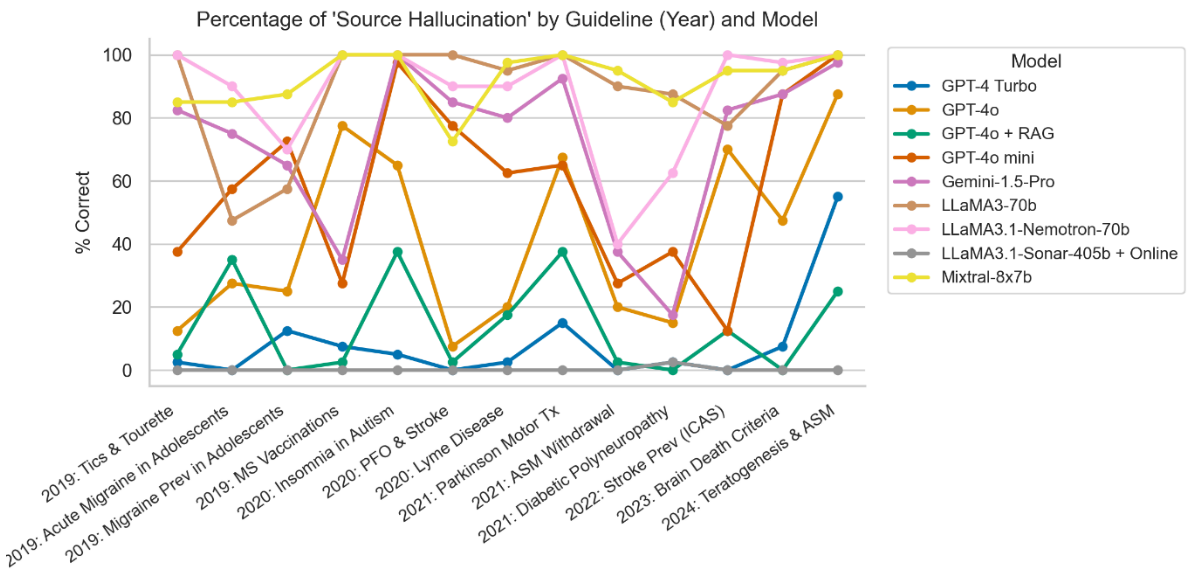

Supplementary Figure 5: Variability in Question Ratings Across Batches by Model
Bar chart comparing the number of questions with differing ratings across various models. All models answered to the same set of 130 guideline related questions four times. Gemini-1.5 Pro shows the highest variation, followed by Mixtral-8x7b and GPT-4o, while LLaMA3.1-Nemotron-70b showed the least variation. GPT-4o showed less variation after being used in a setup with retrieval-augmented generation (RAG).

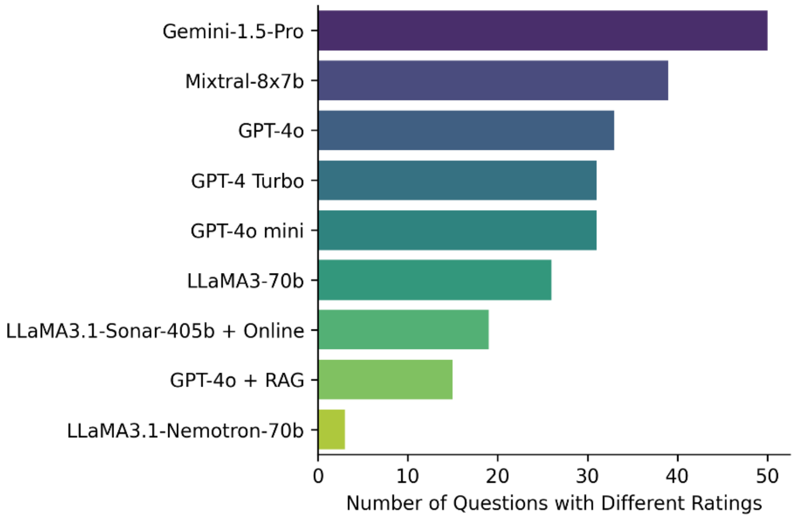

Supplement: Supplementary file 1 — Supplementary Materials [file 41746_2025_1536_MOESM1_ESM.pdf]
